# RETRACTION: Myricetin Possesses Potential Protective Effects on Diabetic Cardiomyopathy through Inhibiting I*κ*B*α*/NF*κ*B and Enhancing Nrf2/HO‐1

**DOI:** 10.1155/omcl/9843608

**Published:** 2026-11-09

**Authors:** Oxidative Medicine and Cellular Longevity

RETRACTION: H‐H. Liao, J‐X. Zhu, H. Feng, et al., “Myricetin Possesses Potential Protective Effects on Diabetic Cardiomyopathy through Inhibiting I*κ*B*α*/NF*κ*B and Enhancing Nrf2/HO‐1,” *Oxidative Medicine and Cellular Longevity*, 2017, 8370593, https://doi.org/10.1155/2017/8370593.

The above article, published online on 24 September 2017 in Wiley Online Library (wileyonlinelibrary.com), and its correction (https://onlinelibrary.wiley.com/doi/10.1155/2021/9812928), has been retracted by agreement the Journal Associate Editor, Janusz Gebicki; and John Wiley & Sons Ltd. The retraction has been agreed due to multiple concerns identified with the Figures, some of which are reported on PubPeer [1]. Further investigation by the publisher identified further concerns. A full summary is below:

• In Figure 4a, the GAPDH bands appear identical to the bottom GAPDH bands shown in Figure 7c.
• In Figure 4b, the Histone 3 bands appear identical to the T‐P65 bands shown in Figure 3d of [2].
• There appears to be unexpected, repeated elements between the M and STZ+M panels of Figure 5a.
• In Figure 5d, the Nuc‐Nrf2 bands appear similar to the RIP2 bands shown in Figure 5a of [3].
• In Figure 5d, the Nrf2 bands appear identical to the Nuc‐Nrf2 bands shown in Figure 5a of [2].
• In Figure 6f, the GAPDH bands are identical to the upper GAPDH bands shown in Figure 7c.
• In Figure 9b, the GAPDH bands appear highly similar to the ß‐Actin bands shown in Figure 5a of [4].
• In Figure 10a, the p‐IκBα bands appear highly similar to the bands shown in lanes 3‐7 of the p‐ACC bands in Figure 4a of [5].

After assessment of the concerns and the author’s responses by the Editorial Board, the results and conclusions can no longer be considered reliable, and the article is therefore retracted.

The authors disagree to the retraction.
